# Premature deaths caused by smoking in Sichuan, Southwest China, 2015–2030

**DOI:** 10.1038/s41598-020-79606-2

**Published:** 2021-01-08

**Authors:** Zhuo Wang, Yu Luo, Shujuan Yang, Kun Zou, Rong Pei, Jun He, Ying Deng, Maigeng Zhou, Li Zhao, Hui Guo

**Affiliations:** 1 Department of Chronic and Non-communicable Disease Control and Prevention, Sichuan Center for Disease Control and Prevention, Chengdu, 610041 China; 2 Key Laboratory of Birth Defects and Related Diseases of Women and Children (Sichuan University), Ministry of Education, West China Second University Hospital, Sichuan University, Chengdu, 610041 China; 3 West China School of Public Health and West China Fourth Hospital, Sichuan University, Chengdu, 610041 Sichuan China; 4 School of Health Caring Industry, Sichuan University of Arts and Science, Dazhou, 635000 Sichuan China; 5 National Center for Chronic and Non-communicable Disease Control and Prevention, Chinese Center for Disease Control and Prevention, Beijing, 100050 China

**Keywords:** Diseases, Risk factors

## Abstract

Smoking has a substantial impact on deaths from non-communicable chronic diseases (NCDs). Quantitatively measuring the impact of tobacco control on population health is of great theoretical and practical importance, for governments to make health policy decisions. Focusing on premature deaths, we predicted the deaths by 2030 from major NCDs caused by smoking among people aged 30–69 years in Sichuan Province, Southwest China. We extracted data for 1990–2015 from the Global Burden of Disease Study 2015 and calculated the population attributable fraction, to estimate the proportion of deaths caused by smoking. Four different tobacco control standards were used to estimate 2030 projections for the prevalence of smoking and premature mortality. If smoking prevalence were reduced by 30% from 2015 levels, premature mortality could be expected to decline by 24.4% in 2030, achieving 81.3% of the World Health Organization target for reducing premature mortality by 30%. Compared with the continuation of historical trends, the strongest tobacco control policy scenario would reduce premature mortality by 6.6%, prevent 23,600 deaths, reduce mortality by 7.8%, and increase life expectancy at birth by 0.3 years. Smoking bans represent an important action toward achieving national health goals.

## Introduction

China is facing a severe chronic disease situation. Of the nearly 10 million deaths occurring in China each year, more than 85% are caused by chronic diseases^1,2^. Smoking is the leading risk factor for chronic diseases in China, leading to nearly 15% of the national disability-adjusted life-years and to more than two million deaths annually^3^.

Sichuan, a province in Southwest China with a developing economy and more than 80 million residents, has higher mortality from both lung cancer and chronic obstructive pulmonary disease than China as a whole, and this difference is thought to be closely related to smoking^4^. According to a 2018 report on population health status and important diseases in Sichuan, 3 of every 10 adults are smokers, 7 of every 10 adult nonsmokers are exposed to secondhand smoke, the smoking rate was 28 times higher among men than among women, and the prevalence rates of smoking and secondhand smoke exposure among men are growing^5^.

The Framework Convention on Tobacco Control (FCTC) is a worldwide campaign launched by the World Health Organization (WHO) in 2003 to create a tobacco-free world^6^. Whereas some high-income countries, such as the United States and the United Kingdom, have shown remarkable declines in smoking prevalence over the past decade, the prevalence of smoking in China has remained very high^7,8^. The WHO estimates that, each year, about six million people worldwide die because of smoking, and most of these deaths occur in low-income and middle-income countries^9^.

Since 2008, Beijing, Shanghai, Hangzhou, Guangzhou, Harbin, Tianjin, Qingdao, Lanzhou, Shenzhen, Changchun, and other cities in China have promulgated local regulations for tobacco control, in accordance with the WHO FCTC guidelines. However, no national regulations have been published, and the implementation of some local laws and regulations lags behind the WHO FCTC requirements^10,11^. In Sichuan Province, there are no local regulations to control smoking. “Healthy China 2030” planning requires the government to promote prohibition of smoking in public places, with the aim to gradually achieve an entirely smoke-free society^12^. To help in achieving the national health goal of “Healthy China 2030” and to help promote the government's tobacco control actions, we predicted premature deaths from major non-communicable chronic diseases (NCDs) among people aged 30–69 years, as well as several other important disease burden indicators in 2030 in Sichuan under four tobacco-control scenarios, based on the smoking prevalence in 2015.

## Methods

### Data source

For the projections in this study, we used data for Sichuan from a collaborative study between the China Center for Chronic Disease Control and Prevention and the Global Burden of Disease Study (GBD) 2015. These data included information on deaths, risk factors, relative risks, covariates (e.g., educational level, urbanization rate, gross domestic product per capita, rainfall, population density, vehicle ownership, medical system availability), and population data by age, sex, and year from 1990 to 2015. More details about the GBD can be found at http://www.healthdata.org/gbd. The predicted population of Sichuan Province in 2030 was obtained from the China Center for Chronic Disease Control and Prevention and the China Population and Development Research Center.

### Four scenarios for tobacco control in 2030

The WHO requires its member states to reduce the current prevalence of tobacco use by 30% by the year 2025 whereas the Chinese government’s target is a reduction of 20% by 2030^12,13^. We created four scenarios to reflect the potential intensity of government action on tobacco control.

- Scenario one—unchanged (Un): The age- and sex-specific prevalence of smoking in 2030 are projected to be the same as in 2015.
- Scenario two—natural trend (NT): The age- and sex-specific prevalence of smoking in 2030 are projected to follow the mean annual rate of change observed from 1990 to 2015.
- Scenario three—Reduced by 20% (R1). The age- and sex-specific prevalence of smoking in 2030 are projected to be 20% lower than the level in 2015.
- Scenario four—reduced by 30% (R2). The age- and sex-specific prevalence of smoking in 2030 are projected to be 30% lower than the level in 2015.

### Projection of NCD premature mortality caused by smoking in 2030

As many as 15 million people die annually from chronic diseases between the ages of 30 and 69 years, and many of these deaths can be avoided. The WHO defines death among people aged 30 to < 70 years as premature death, and this indicator is used to evaluate the ability of countries to prevent and control chronic diseases^14^. We used the following three steps to estimate the NCD premature mortality attributable to smoking in Sichuan in 2030, according to disease, age (in 5-year groups), and sex.

- Step one: We estimated the prevalence of smoking in 2030. Direct and indirect methods were used to predict smoking exposure. In the direct method, we used the current and former prevalence to measure exposure. This method was used in estimating the mortality from diseases such as tuberculosis, lower respiratory tract infection, and ischemic heart disease, which are not strongly related to daily smoking or age at smoking initiation. The formula is as follows:
  $$ {\text{Exposure}}_{2030} = {\text{exposure}}_{2015} {\text{*exp}}\left( {\frac{{{\text{In}}\left( {\frac{{{\text{exposure}}_{2015} }}{{{\text{exposure}}_{1990} }}} \right)}}{2015 - 1990}{*}\left( {2030 - 2015} \right)} \right) $$

The indirect method uses the smoking impact ratio (SIR) as the exposure, which indirectly reflects the level of smoking exposure in the population. The SIR was used to estimate the mortality from esophageal cancer, lung cancer, chronic obstructive pulmonary disease, and other diseases that are strongly correlated with cumulative harm from tobacco^15^. The formula for calculating the SIR is as follows:

$$ {\text{SIR}} = \frac{{{\text{C}}_{{{\text{LC}}}} - {\text{N}}_{{{\text{LC}}}} }}{{{\text{S}}_{{{\text{LC}}}}^{*} - {\text{N}}_{{{\text{LC}}}}^{*} }}{*}\frac{{{\text{N}}_{{{\text{LC}}}}^{*} }}{{{\text{N}}_{{{\text{LC}}}} }}, $$

where C_LC_ is the age-sex specific lung cancer mortality rate of the population in this study, and N_LC_ is the age-sex specific lung cancer mortality rate of nonsmokers in the present study population. S*_LC_ and N*_LC_ represent the age- and sex-specific lung cancer mortality rates of the known standard reference population of smokers and nonsmokers, respectively. The values for S*_LC_ and N*_LC_ were obtained from the American Cancer Society Cancer Prevention Study II^16^, and that for N_LC_ was from the China Kadoorie Biobank study^17^_._

(b) Step two: We calculated the population attributable fraction (PAF) of smoking. Here, we categorized all deaths into those that were attributable to smoking and those that were not attributable to smoking, according to the theory of comparative risk assessment^18^. The formula for the PAF is as follows:
  $$ {\text{ PAF}} = \frac{{\mathop \sum \nolimits_{{{\text{i}} = 1}}^{{\text{n}}} {\text{P}}_{{\text{i}}} \left( {{\text{RR}}_{{\text{i}}} - 1} \right)}}{{\mathop \sum \nolimits_{{{\text{i}} = 1}}^{{\text{n}}} {\text{P}}_{{\text{i}}} \left( {{\text{RR}}_{{\text{i}}} - 1} \right) + 1}} $$
(c) Step three: The life-table method was used to estimate the probability of death owing to NCDs between age 30 and 69 years according to age-specific death rates (in 5-year age groups from 30 to < 70 years)^19^. The formula for calculating premature mortality is as follows:
  $$ { }{}_{40}^{{}} {\text{q}}_{30} = 1 - \mathop \prod \limits_{{{\text{x}} = 30}}^{65} \left( {1 - {}_{5}^{{}} {\text{q}}_{{\text{x}}} } \right) $$

### Effect of tobacco control strategy on disease burden

In addition to premature mortality, the number of deaths, mortality rate, and life expectancy were also analyzed under the four scenarios of tobacco control.

### Ethical approval

Not required.

## Results

### Smoking prevalence in Sichuan

Table 1 presents the prevalence of smoking under the four projected scenarios. The prevalence of smoking was 12 times higher for men than for women. Men aged 50–54 years had the highest smoking rate, whereas the smoking rate for women gradually increased with age. Under the different tobacco intervention scenarios, the smoking rates in 2030 among men were 43.8% (Un), 35.8% (NT), 35.1% (R1), and 30.7% (R2). For women, the projected 2030 smoking rates were 3.5% (Un), 3.1% (NT), 2.8% (R1), and 2.5% (R2). Compared with the unchanged situation (Un), under the other three scenarios, the smoking rate in 2030 was projected to decrease by 18.3% (NT), 19.9% (R1), and 29.9% (R2) among men and by 11.4% (NT), 20.0% (R1), and 28.6% (R2) among women. The SIR was projected to increase by 6.8% (NT), decrease by 20.3% (R1), and decrease by 30.1% (R2) among men and to decline by 38.8% (NT), 20.4% (R1), and 30.6% (R2) among women.

**Table 1:** Age- and sex-specific smoking exposure in 2030 under four scenarios.

Gender	Age	Prevalence of smoking (%)				SIR			
		Un	NT	R1	R2	Un	NT	R1	R2
Men	15 ~	22.5	23.6	18.0	15.7	–	–	–	–
	20 ~	41.3	34.9	33.0	28.9	–	–	–	–
	25 ~	47.0	38.6	37.6	32.9	–	–	–	–
	30 ~	45.6	34.4	36.5	32.0	13.1	10.3	10.5	9.2
	35 ~	48.5	38.9	38.8	33.9	13.1	10.3	10.5	9.2
	40 ~	50.4	41.4	40.3	35.3	13.1	10.3	10.5	9.2
	45 ~	50.3	41.9	40.2	35.2	34.4	33.6	27.5	24.1
	50 ~	51.0	41.3	40.8	35.7	18.0	20.9	14.4	12.6
	55 ~	50.1	40.9	40.1	35.1	12.1	15.4	9.7	8.5
	60 ~	44.9	36.0	35.9	31.4	11.6	16.1	9.3	8.2
	65 ~	40.4	31.1	32.3	28.3	9.2	13.3	7.4	6.5
	70 ~	38.7	31.0	30.9	27.1	6.7	8.2	5.4	4.7
	75 ~	32.1	25.0	25.7	22.5	5.1	6.8	4.1	3.6
	80 ~	30.7	28.8	24.6	21.5	5.1	6.8	4.1	3.6
	Total	43.8	35.8	35.1	30.7	13.3	14.2	10.6	9.3
Women	15 ~	1.2	1.1	0.9	0.8	–	–	–	–
	20 ~	1.6	1.0	1.3	1.1	–	–	–	–
	25 ~	2.2	1.8	1.8	1.5	–	–	–	–
	30 ~	1.6	0.8	1.3	1.1	18.0	5.9	14.4	12.6
	35 ~	1.9	1.0	1.5	1.3	18.0	5.9	14.4	12.6
	40 ~	3.6	3.5	2.9	2.5	18.0	5.9	14.4	12.6
	45 ~	2.6	1.6	2.1	1.8	8.2	5.2	6.6	5.7
	50 ~	3.8	2.4	3.0	2.6	11.6	10.6	9.3	8.1
	55 ~	3.7	1.6	2.9	2.6	7.2	6.0	5.7	5.0
	60 ~	3.2	1.3	2.5	2.2	7.6	7.2	6.1	5.3
	65 ~	6.0	6.9	4.8	4.2	4.8	5.1	3.9	3.4
	70 ~	5.2	4.9	4.2	3.7	3.7	4.9	2.9	2.6
	75 ~	6.5	9.7	5.2	4.6	1.9	4.0	1.5	1.3
	80 ~	7.1	9.4	5.7	5.0	1.9	4.0	1.5	1.3
	Total	3.5	3.1	2.8	2.5	9.8	6	7.8	6.8

*Un* unchanged scenario, *NT* natural trend scenario, *R1* reduced by 20% scenario, *R2* reduced by 30% scenario, *SIR* smoking impact ratio.

### Number of deaths and mortality rates for the main NCDs at ages 30–69 years, 1990–2030

Table 2 and Table S1 show the number of NCD deaths, the mortality rate for those aged 30–69 years from 1990 to 2030, as well as the relative changes of deaths and mortality in 2030 under different scenarios, as compared with 2015. Following historic trends, the total deaths and mortality rate from NCDs in 2030 for people aged 30–69 were projected to be 266,900 and 540.0 per 100,000, respectively. Compared with 2015, the number of deaths under the different scenarios was projected to increase by 49,500 (Un), increase by 400 (NT), decrease by 16,800 (R1), and decrease by 23,200 (R2). The mortality rate was projected to increase by 5.1% (Un), decrease by 11.3% (NT), decrease by 17.0% (R1), and decrease by 19.1% (R2). The ranking of the four scenarios from largest to smallest in terms of the reduction in mortality is as follows: R2, R1, NT, and Un. The effect of the tobacco control policy under R2 could prevent 23,600 deaths and reduce the mortality rate by 7.8%, compared with the NT scenario.

**Table 2:** Deaths and mortality owing to the main NCDs for people aged 30–69 years from 2015 to 2030 under different scenarios.

Gender	Diseases	2015		2030								Reduction in deaths (in thousands)^a^				Percent change of Mortality rate (%)^a^			
				Un		NT		R1		R2									
		Deaths (in thousands)	Mortality rate (per 100,000)	Deaths (in thousands)	Mortality rate (per 100,000)	Deaths (in thousands)	Mortality rate (per 100,000)	Deaths (in thousands)	Mortality rate (per 100,000)	Deaths (in thousands)	Mortality rate (per 100,000)	Un	NT	R1	R2	Un	NT	R1	R2
Both	Total	266.4	608.7	316.0	639.4	266.9	540.0	249.6	505.1	243.2	492.2	49.5	0.4	− 16.8	− 23.2	5.1	− 11.3	− 17.0	− 19.1
	Cancer	117.7	268.8	138.3	280.0	133.1	269.3	120.3	243.4	117.3	237.3	20.7	15.4	2.6	− 0.4	4.1	0.2	− 9.5	− 11.7
	CVD	75.8	173.2	93.4	189.1	77.8	157.4	77.9	157.6	75.5	152.9	17.6	1.9	2.0	− 0.3	9.1	− 9.1	− 9.0	− 11.7
	CRD	40.2	91.8	50.2	101.7	23.7	47.9	19.2	38.8	18.1	36.7	10.1	− 16.5	− 21.0	− 22.1	10.8	− 47.8	− 57.8	− 60.0
	DM	4.7	10.8	5.9	12.0	4.3	8.7	4.3	8.7	4.3	8.6	1.2	− 0.4	− 0.4	− 0.5	11.6	− 19.4	− 19.3	− 20.1
Men	Total	178.3	810.0	210.3	844.5	186.5	748.8	170.2	683.3	164.8	661.9	32.0	8.2	− 8.1	− 13.5	4.3	− 7.6	− 15.6	− 18.3
	Cancer	80.9	367.6	95.6	384.0	95.6	383.7	83.2	334.2	80.8	324.4	14.7	14.6	2.3	− 0.1	4.5	4.4	− 9.1	− 11.8
	CVD	48.8	221.5	59.4	238.4	51.7	207.7	51.8	207.9	49.7	199.4	10.6	3.0	3.0	0.9	7.6	− 6.2	− 6.1	− 10.0
	CRD	26.4	119.9	32.5	130.6	17.0	68.3	13.0	52.0	12.2	49.1	6.1	− 9.4	− 13.4	− 14.2	8.9	− 43.1	− 56.6	− 59.0
	DM	2.3	10.6	2.9	11.5	2.3	9.2	2.3	9.2	2.3	9.1	0.5	0.0	0.0	− 0.1	8.7	− 13.1	− 13.0	− 14.5
Women	Total	88.1	405.0	105.7	431.1	80.4	328.0	79.4	324.1	78.4	319.8	17.5	− 7.7	− 8.7	− 9.7	6.4	− 19.0	− 20.0	− 21.0
	Cancer	36.8	168.9	42.7	174.2	37.5	153.0	37.0	151.1	36.5	148.9	5.9	0.8	0.3	− 0.3	3.1	− 9.4	− 10.6	− 11.9
	CVD	27.1	124.4	34.1	138.9	26.1	106.4	26.1	106.4	25.9	105.7	7.0	− 1.0	− 1.0	− 1.2	11.6	− 14.5	− 14.5	− 15.1
	CRD	13.8	63.3	17.7	72.3	6.7	27.3	6.2	25.3	5.9	24.0	3.9	− 7.1	− 7.6	− 7.9	14.1	− 56.9	− 60.1	− 62.1
	DM	2.4	11.0	3.1	12.6	2.0	8.2	2.0	8.2	2.0	8.2	0.7	− 0.4	− 0.4	− 0.4	14.5	− 25.5	− 25.5	− 25.6

*CVD* cardiovascular diseases, *CRD* chronic respiratory diseases, *DM* diabetes mellitus, *Un* unchanged scenario, *NT* natural trend scenario, *R1* reduced by 20% scenario, *R2* reduced by 30% scenario, *SIR* smoking impact ratio.

^a^Compared with 2015.

For the four main chronic diseases, the impact of tobacco control on the death burden varied. Cancer caused 117,700 deaths in 2015, and this number was projected to be 138,300, 133,100, 120,300, and 117,300 in 2030 under the Un, NT, R1, and R2 scenarios, respectively. The cancer mortality rate of 268.8 per 100,000 in 2015 was projected to be 280.0 (Un), 269.3 (NT), 243.4 (R1), and 237.3 (R2) per 100,000 in 2030, reflecting an increase of 4.1% (Un), an increase of 0.2% (NT), a decrease of 9.5% (R1), and a decrease of 11.7% (R2), as compared with 2015. Cardiovascular disease (CVD) mortality was projected to increase by 9.1% (Un), decrease by 9.1% (NT), decrease by 9% (R1), and decrease by 11.7% (R2). Mortality owing to chronic respiratory diseases (CRD) was projected to increase by 10.8% (Un), decrease by 47.8% (NT), decrease by 57.8% (R1), and decrease by 60.0% (R2). Diabetes mellitus (DM) mortality was projected to increase by 11.6% (Un), decrease by 19.4% (NT), decrease by 19.3% (R1), and decrease by 20.1% (R2) (Table 2).

The analysis by sex showed that tobacco control would have a large effect among both men and women. For men, a reduction of more than 13,000 deaths and an 18.3% decline in the mortality rate, compared with the situation in 2015, were projected under the R2 scenario. Again considering the R2 scenario, there would be 9,700 fewer deaths and a 21% reduction in the mortality rate for women.

Compared with the continuation of historical trends, tobacco controls would reduce the number of deaths, as follows. Under the R2 scenario, there would be an annual reduction of 16,000 cancer deaths, 6000 CRD deaths, and 2000 CVD deaths, compared with the NT scenario. Compared with 2015, the largest decrease was projected for the CRD mortality rate, which would be reduced by 60%. The cancer, CVD, and DM mortality rates would also be reduced (by 11.7%, 11.7%, and 20.1%, respectively) if the age- and sex-specific prevalence of smoking were 30% lower in 2030 than in 2015 (R2).

### Improvement in premature mortality under different tobacco control scenarios

Table 3 and Figure S2 present our projections of premature mortality from the main NCDs (namely, CVD, cancer, CRD, and DM) among people aged 30–69 years from 2015 to 2030 under the four different scenarios, as well as comparisons of the benefits of different tobacco control strategies. Compared with 2015, the probability of dying between age 30 and 69 years would be minimized under the R2 scenario. In 2030, premature mortality caused by NCDs would be 23.6%, 19.4%, 18.2%, and 17.8% under the Un, NT, R1, and R2 scenarios, respectively, and total premature mortality would be reduced by 24.4% if the smoking prevalence in 2030 were reduced by 30% (R2) (Table 3). Compared with the continuation of historical trends, the tobacco control policy under the R2 scenario could result in an additional reduction in premature mortality of 6.6%.

**Table 3:** Premature mortality (%) of the main NCDs for people aged 30–69 years from 2015 to 2030.

Gender	Diseases	2015	2030				Percent change^a^ (%)			Proportion of target completion^b^ (%)		
			Un	NT	R1	R2	NT	R1	R2	NT	R1	R2
Both	Total	23.6	23.6	19.4	18.2	17.8	− 17.8	− 22.7	− 24.4	59.3	75.7	81.3
	Cancer	11.1	11.1	10.1	9.2	9.0	− 8.8	− 17.2	− 19.2	29.3	57.3	64.0
	CVD	7.4	7.4	6.1	6.1	6.0	− 17.4	− 17.4	− 19.7	58.0	58.0	65.7
	CRD	4.1	4.1	1.9	1.6	1.5	− 52.5	− 61.6	− 63.6	175.0	205.3	212.0
	DM	0.5	0.5	0.3	0.3	0.3	− 27.3	− 27.3	− 28.0	91.0	91.0	93.3
Men	Total	28.9	28.9	26.1	24.1	23.5	− 9.7	− 16.5	− 18.8	32.3	55.0	62.7
	Cancer	14.3	14.3	14.2	12.5	12.2	− 0.1	− 12.1	− 14.5	0.3	40.3	48.3
	CVD	9.2	9.2	8.1	8.1	7.8	− 12.1	− 11.9	− 15.3	40.3	39.7	51.0
	CRD	5.2	5.2	2.8	2.1	2.0	− 46.5	− 59.1	− 61.4	155.0	197.0	204.7
	DM	0.5	0.5	0.4	0.4	0.4	− 19.8	− 19.8	− 21.1	66.0	66.0	70.3
Women	Total	15.6	15.6	12.1	12.0	11.8	− 22.4	− 23.4	− 24.4	74.7	78.0	81.3
	Cancer	6.6	6.6	5.8	5.7	5.7	− 11.8	− 12.9	− 14.1	39.3	43.0	47.0
	CVD	5.4	5.4	4.1	4.1	4.1	− 22.9	− 23.2	− 23.7	76.3	77.3	79.0
	CRD	2.8	2.8	1.1	1.0	1.0	− 61.7	− 64.6	− 66.3	205.7	215.3	221.0
	DM	0.5	0.5	0.3	0.3	0.3	− 34.6	− 34.6	− 34.6	115.3	115.3	115.3

*CVD* cardiovascular diseases, *CRD* chronic respiratory diseases, *DM* diabetes mellitus, *Un* unchanged scenario, *NT* natural trend scenario, *R1* reduced by 20% scenario, *R2* reduced by 30% scenario, *SIR* smoking impact ratio.

^a^Compared with 2015.

^b^Proportion of target completion was calculated by dividing percent change by 30%, which was compared with the World Health Organization standard (30% lower than 2015).

In each NCD subcategory, men generally had higher premature mortality than did women by 2030, and the decline in premature mortality owing to tobacco control policy under the different tobacco control scenarios was more substantial among men than among women. The largest decline was seen in premature mortality caused by CRD, which was projected to drop by 63.6% in the R2 scenario, compared with the unchanged rate (Un). Under the same scenario, cancer, CVD, and DM mortality would decline by 19.2%, 19.7%, and 28.0%, respectively. The ranking of diseases according to the size of decline was the same for men and women.

The WHO requires its member states to achieve a global target of a one-third reduction from current levels in premature mortality owing to NCDs by 2030^20^. We show the target line of Sichuan Province in Figure S2, whose premature mortality was 23.6% in 2015; the target value was 16.5% by 2030. Target completion can be assessed in Table 3. Through the three different tobacco control scenarios of NT, R1, R2, we can achieve 59.3%, 75.5%, and 81.3% of the WHO requirements, respectively.

### Life expectancy at birth and population pyramid in Sichuan from 2015 to 2030

**Figure 1:**
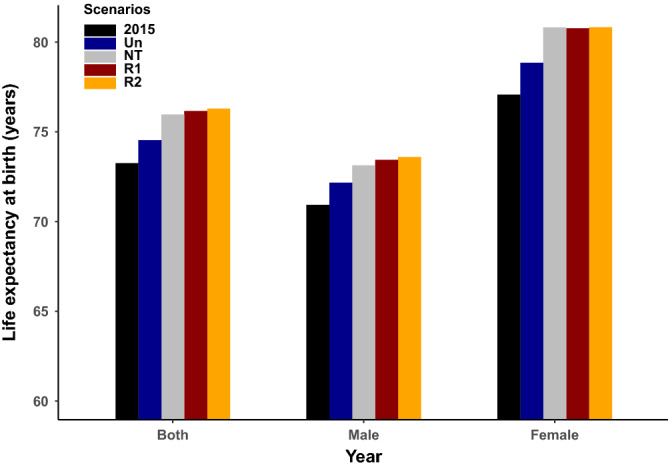
Life expectancy at birth in Sichuan in 2015 and under four scenarios by 2030. *Un* unchanged scenario, *NT* natural trend scenario, *R1* reduced by 20% scenario, *R2* reduced by 30% scenario.

The contribution of tobacco control to life expectancy of the total population is shown in Fig. 1. Compared with 2015, life expectancy at birth under the Un, NT, R1, and R2 scenarios was projected to increase by 1.3, 2.7, 2.9, and 3 years, respectively. Under the R2 scenario, men’s life expectancy was projected to increase more than women’s life expectancy.

Figure S1 shows the Sichuan population pyramid in 2015 and 2030, with a larger aging population structure in 2030.

## Discussion

To date, Sichuan Province in Southwest China has not promulgated local regulations to control smoking, and the high smoking rate among the population has not changed. Our research demonstrated in detail the importance of tobacco control policies for people’s health. Through 2030, if the prevalence of smoking can be reduced by 30% in comparison with 2015, premature mortality is expected to decline by 24.4%, achieving 81.3% of the WHO requirement. Under this same tobacco control scenario, deaths caused by NCDs among those aged 30–69 years would decline by 23,200, mortality would decrease by 19.1%, and life expectancy at birth would increase by 3 years. Compared with a continuation of historical trends, the most stringent tobacco control policy (R2) would lead to a 6.6% reduction in premature mortality, the prevention of 23,600 deaths, a 7.8% decline in mortality, and a 0.3 year increase in life expectancy at birth.

Preventing and controlling chronic diseases are serious public health concerns globally^21^. Interventions to combat risk factors are among the most important measures to reduce the disease burden, and governments play a large role in these interventions. In a 2018 political declaration, the United Nations (UN) recognized that existing governmental actions cannot reduce premature mortality and disability risks sufficiently to meet the Sustainable Development Goals^22^. The UN therefore put forward five prevention and control strategies to combat five major risk factors, namely, controlling an unhealthy diet, tobacco use, air pollution, harmful alcohol consumption, and lack of physical activity. In China, the prevalence of smoking is high. The “Report on the Health Hazards of Smoking in China,” published by the Ministry of Health in 2012, pointed out that China is the largest producer and consumer of tobacco in the world, with more than 300 million smokers and about 740 million people exposed to secondhand smoke^23^. We can see from Tables 2 and 3 that there are many opportunities for the government to contribute to people’s health in this context. Even considering population aging (Figure S1), government interventions can still serve to reduce the number of deaths and mortality and to improve life expectancy. For example, if historical trends continue, the change in premature mortality will reach 59.3% of the WHO target, and a tobacco control policy can result in the achievement of another 22% of this target. To fully meet the WHO target, interventions controlling other risk factors will also need to be implemented.

Smoking and secondhand smoke exposure are risk factors for many diseases^24,25^. The relationships between smoking exposure and lung cancer, respiratory diseases, and other diseases were officially reported as early as 1986 in the United States Surgeon General reports^26^. However, public awareness of the hazards of smoking and secondhand smoke exposure remains inadequate. This can be observed from the smoking rate in Sichuan, which was 43.8% among men and 3.5% among women in 2015. Based on a report by Li (2017), the smoking prevalence among men in Sichuan is similar to that in China, and this prevalence among women in Sichuan is higher than elsewhere in China^27^. If historical trends continue, 35.8% of men and 3.1% of women will smoke in 2030. It is our view that the government has not done enough to change smoking behavior in the population. One reason for this may be a lack of attention to this issue on the part of the government.

Some politicians and powerful economic entities have argued that policies forbidding tobacco use could hinder economic development by reducing income from taxes^28,29^. However, accumulated international experience has shown that strict tobacco cessation can result in considerable improvement in people’s health without restricting economic development, and the advantages outweigh the disadvantages in the long run^30–32^. People aged 30–69 years are the main labor force in society and also make up the largest group of smokers. Serious labor losses result if many people die from chronic diseases in their 30 s or 40 s. The WHO has proposed using reductions in premature mortality to reflect the effect of chronic disease prevention and control. Implementing measures to ban tobacco use in Sichuan Province would bring enormous health benefits to the labor force. Under the R2 scenario examined in this study, the number of deaths would be reduced by 22,000 per year among men, including the prevention of 15,000 cancer deaths, 5000 CRD deaths, and 2000 CVD deaths, compared with the NT scenario. The largest reduction in the mortality rate would be seen for CRD. In addition to having a direct impact on smoking, smoking control measures would also lead to a decline in the exposure to secondhand smoke among the entire population, which would result in additional health benefits for nonsmokers.

In many high-level discussions, many strategies and measures have been proposed to fight chronic diseases^33–35^. If we were to select only one action, tobacco control interventions may be the best choice at present in Sichuan Province, Southwest China. Several cost-effective actions, such as raising taxes, using large pictorial health warnings, and banning smoking in public places, have not yet been sufficiently implemented in China^27,36^. As reported by Foreman (2018), Lozano (2018), and other researchers, the global average smoking rate will decrease each year in the future; however, the smoking prevalence will remain high in East Asia and tobacco will become the most important risk factor for chronic diseases in East Asia^37,38^. Taking action to implement tobacco control changes in Sichuan can serve as a good example for China and the rest of the world.

### Strengths and limitations

Smoking has a substantial impact on deaths caused by NCDs. Quantitative measurement of the impact of tobacco control on population health is of great theoretical and practical importance for governments seeking to formulate disease prevention and control strategies and to achieve the “Healthy China 2030” goal. To the best of our knowledge, there have been few previous reports on tobacco control strategies in Southwest China^39^. The economic level and health awareness in this region lag behind the rest of the country, making additional policy support from local governments necessary. In the present study, we calculated projections based on the GBD method, and our estimates of premature mortality and life expectancy can be compared directly with results from around the world. We examined four different scenarios, to clearly show the different projected future consequences of these scenarios, including no change, the continuation of historical trends, and the application of policies with different strengths.

Our study has several limitations. First, the estimation was based on original GBD data, which may have led to underestimation or overestimation of the effects. For example, the associations between smoking and the examined diseases might be different for the Chinese population than what is seen in the global data. Second, many important health-related risk factors, such as poor diet and lack of physical activity, were not adjusted in this study, and we assumed that their trends would remain unchanged. The interactions between these omitted variables were therefore ignored, which could be one reason for bias in estimating the burden of death. However, this did not influence our quantitative assessment and projected results of tobacco control. As a next step, we plan to include more factors, to provide additional evidence for policy decision making. In general, it would be preferable to take the step of implementing tobacco control as soon as possible, to begin working toward achieving the WHO requirements.

## Conclusions

If the prevalence of smoking were reduced by 30%, premature mortality caused by NCDs would be 24.4% lower in 2030 than in 2015. Actions of the government in Southwest China to formulate or implement smoking ban legislation can greatly improve public health by reducing smoking. By providing a quantitative assessment of the potential changes in deaths, our findings can encourage governments, society, and individuals, to pay greater attention to the problem of smoking.

## Supplementary Information

Supplementary Information

## Data Availability

The data that support our study are available from Sichuan CDC and CCDC but restrictions apply to the availability of these data. National data can be accessed from the following link in public: https://gbd2016.healthdata.org/gbd-search/, but Provincial data were not available in public. Data are however available from the authors on reasonable request.
