# Supplementary material for: Premature deaths caused by smoking in Sichuan, Southwest China, 2015–2030
Source: Sci Rep. 2021 Jan 8;11:171. doi: 10.1038/s41598-020-79606-2 (PMC7794491; doi:10.1038/s41598-020-79606-2)
Supplement: Supplementary file 1 — Supplementary Information [file 41598_2020_79606_MOESM1_ESM.docx]

Premature deaths caused by smoking in Sichuan, Southwest China, 2015–2030

Zhuo Wang^1#^, Yu Luo^2#^, Shujuan Yang^3^, Kun Zou^3^, Rong Pei^4^, Jun He^1^, Ying Deng^1^, Maigeng Zhou^5^, Li Zhao^3*^, Hui Guo^2*^

1 Department of Chronic and Non-communicable Disease Control and Prevention, Sichuan Center for Disease Control and Prevention, Chengdu 610041, China.

2 Key Laboratory of Birth Defects and Related Diseases of Women and Children (Sichuan University), Ministry of Education, West China Second University Hospital, Sichuan University, Chengdu 610041, China.

3 West China School of Public Health and West China Fourth Hospital, Sichuan University, Chengdu, Sichuan 610041, China.

4 School of Health Caring Industry, Sichuan University of Arts and Science, Dazhou, Sichuan 635000, China

5 National Center for Chronic and Non-communicable Disease Control and Prevention, Chinese Center for Disease Control and Prevention, Beijing 100050, China.

^#^Contributed equally.

*Correspondence: Li Zhao, email: [zhaoli@scu.edu.cn](mailto:zhaoli@scu.edu.cn);

Hui Guo, email: 491162470@qq.com

**Supplemental materials**

Additional file 1. Additional tables.

Table S1. Deaths number, crude mortality, premature mortality of main NCDs for people aged 30-69 from 1990 to 2010 in Sichuan

| Gender | Disease ^a^ | 1990 | | | 1995 | | | 2000 | | | 2005 | | | 2010 | | |
| --- | --- | --- | --- | --- | --- | --- | --- | --- | --- | --- | --- | --- | --- | --- | --- | --- |
|  |  | Deaths (thousands) | Death rate (per 100,000) | Premature mortality (%) | Deaths (thousands) | Death rate (per 100,000) | Premature mortality (%) | Deaths (thousands) | Death rate (per 100,000) | Premature mortality (%) | Deaths (thousands) | Death rate (per 100,000) | Premature mortality (%) | Deaths (thousands) | Death rate (per 100,000) | Premature mortality (%) |
| Both | Total | 308.6 | 757.7 | 37.2 | 285.1 | 710.2 | 34.8 | 268.6 | 673.8 | 33.8 | 270.6 | 634.0 | 30.8 | 254.9 | 582.0 | 26.2 |
|  | Cancer | 114.6 | 281.4 | 15.1 | 111.8 | 278.4 | 14.7 | 113.5 | 284.6 | 15.2 | 118.7 | 278.1 | 14.3 | 115.4 | 263.6 | 12.5 |
|  | CVD | 80.5 | 197.8 | 11.6 | 68.0 | 169.3 | 9.8 | 64.1 | 160.8 | 9.5 | 69.5 | 162.8 | 9.1 | 71.2 | 162.7 | 8.2 |
|  | CRD | 82.1 | 201.5 | 12.3 | 73.8 | 183.7 | 11.2 | 58.7 | 147.2 | 9.3 | 49.8 | 116.7 | 7.1 | 35.8 | 81.8 | 4.5 |
|  | DM | 3.3 | 8.2 | 0.5 | 3.6 | 9.0 | 0.5 | 4.3 | 10.8 | 0.7 | 4.6 | 10.7 | 0.6 | 4.3 | 9.9 | 0.5 |
| Men | Total | 186.2 | 869.7 | 38.5 | 176.2 | 841.0 | 36.5 | 168.0 | 815.6 | 35.5 | 172.8 | 790.0 | 33.6 | 168.1 | 756.7 | 30.3 |
|  | Cancer | 73.4 | 342.8 | 16.3 | 71.8 | 342.6 | 15.9 | 73.2 | 355.2 | 16.5 | 78.4 | 358.2 | 16.4 | 78.4 | 352.6 | 15.2 |
|  | CVD | 46.5 | 217.2 | 12.5 | 40.7 | 194.2 | 10.8 | 38.3 | 185.9 | 10.4 | 42.4 | 193.6 | 10.3 | 44.9 | 201.9 | 9.8 |
|  | CRD | 44.9 | 209.9 | 12.9 | 42.2 | 201.4 | 12.2 | 34.6 | 168.2 | 10.4 | 30.0 | 137.3 | 8.1 | 23.0 | 103.4 | 5.5 |
|  | DM | 1.5 | 6.9 | 0.4 | 1.6 | 7.9 | 0.5 | 2.0 | 9.7 | 0.6 | 2.2 | 9.9 | 0.6 | 2.1 | 9.3 | 0.5 |
| Women | Total | 122.4 | 633.6 | 29.4 | 108.9 | 567.4 | 26.2 | 100.6 | 522.3 | 24.8 | 97.8 | 469.9 | 22.0 | 86.7 | 402.0 | 17.6 |
|  | Cancer | 41.2 | 213.5 | 9.7 | 40.0 | 208.2 | 9.3 | 40.3 | 209.1 | 9.6 | 40.3 | 193.8 | 8.9 | 37.1 | 171.9 | 7.4 |
|  | CVD | 34.1 | 176.3 | 10.0 | 27.3 | 142.0 | 7.9 | 25.8 | 134.1 | 7.7 | 27.1 | 130.4 | 7.3 | 26.4 | 122.3 | 6.2 |
|  | CRD | 37.1 | 192.2 | 11.5 | 31.6 | 164.5 | 9.9 | 24.1 | 124.8 | 7.9 | 19.8 | 95.1 | 5.8 | 12.9 | 59.6 | 3.3 |
|  | DM | 1.9 | 9.6 | 0.6 | 2.0 | 10.3 | 0.6 | 2.3 | 12.1 | 0.7 | 2.4 | 11.6 | 0.7 | 2.3 | 10.5 | 0.6 |

a CVD-cardiovascular diseases; CRD-chronic respiratory diseases; DM-diabetes mellitus

Additional file 2. Additional figures.

| A 2015 | B 2030 |
| --- | --- |
| 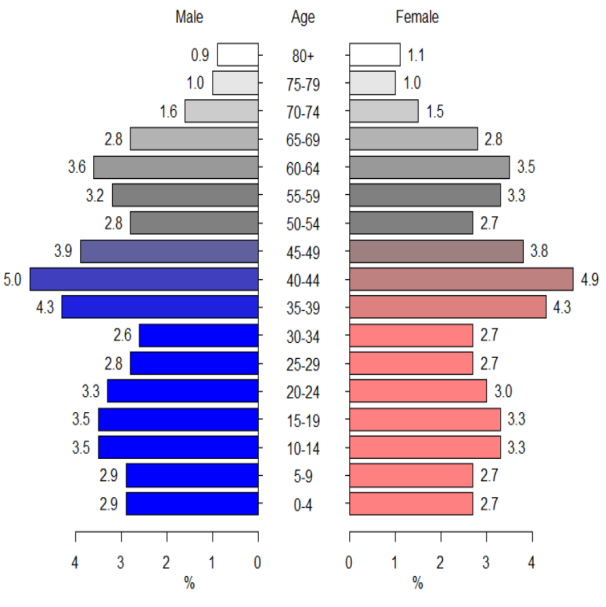 | 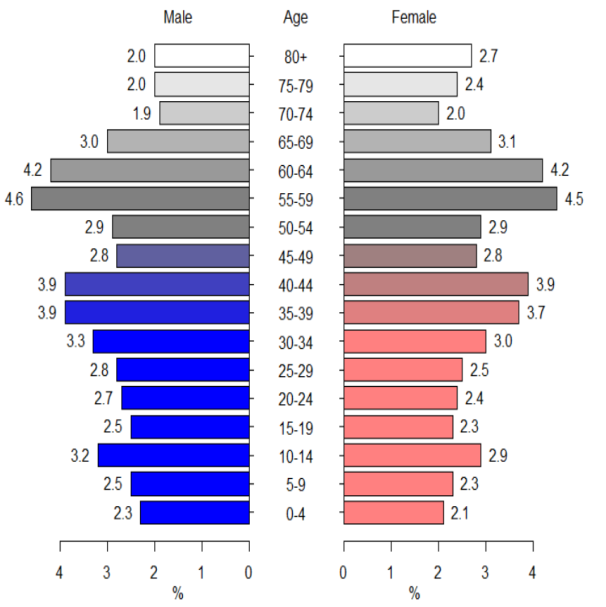 |
| Figure S1 Sichuan population pyramid. | |

| 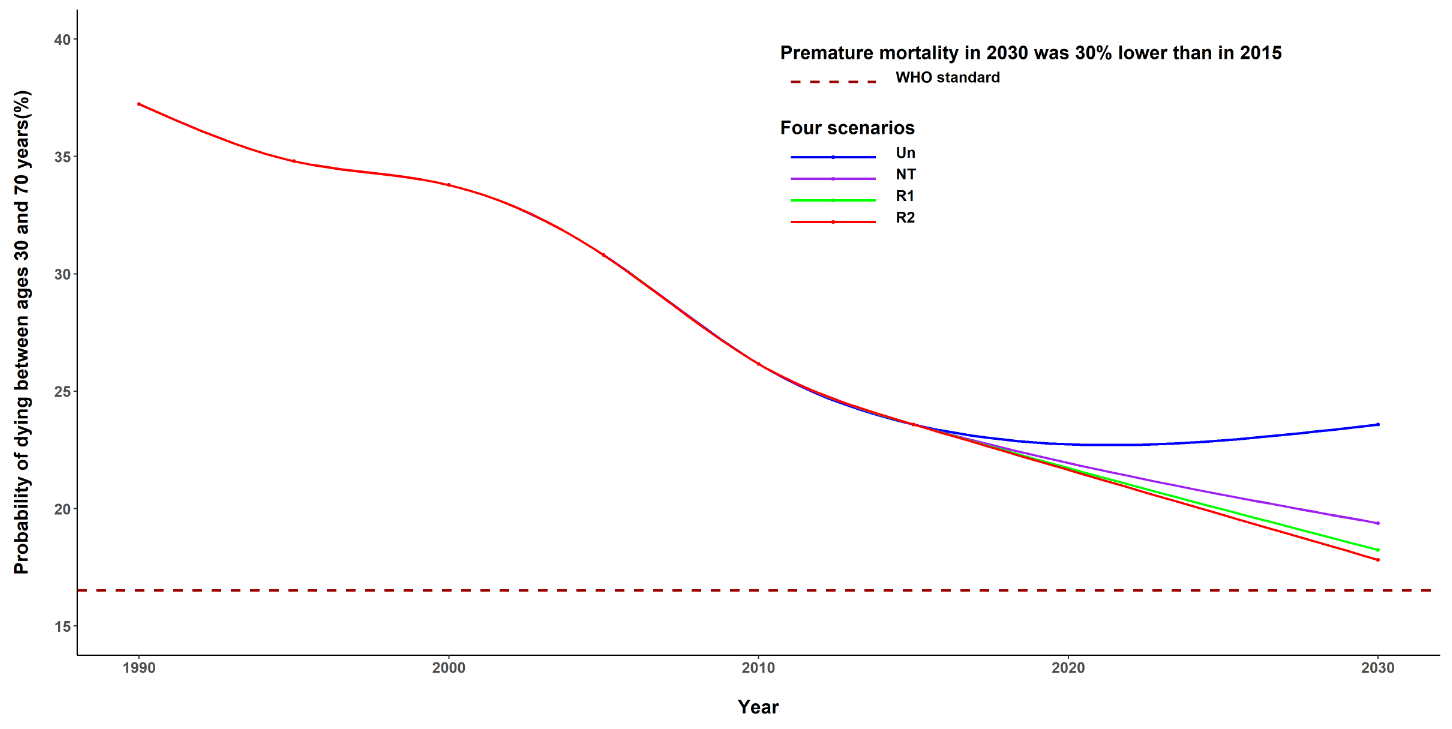 |
| --- |
| Figure S2 Probability of premature death owing to non-communicable chronic diseases among people aged 30–69 in Sichuan from 1990 to 2030.  Un, unchanged scenario; NT, natural trend scenario; R1, reduced by 20% scenario; R2, reduced by 30% scenario. |
